# TCR activation mimics CD127^low^PD-1^high^ phenotype and functional alterations of T lymphocytes from septic shock patients

**DOI:** 10.1186/s13054-018-2305-5

**Published:** 2019-04-17

**Authors:** Julie Mouillaux, Camille Allam, Morgane Gossez, Thomas Uberti, Benjamin Delwarde, Jack Hayman, Thomas Rimmelé, Julien Textoris, Guillaume Monneret, Estelle Peronnet, Fabienne Venet

**Affiliations:** 1 EA 7426 « Pathophysiology of injury-induced immunosuppression (PI3) » Lyon 1 University / Hospices Civils de Lyon / bioMérieux, Hôpital Edouard Herriot 5 place d’Arsonval, 69003 Lyon, France; 2 Joint Research Unit HCL-bioMérieux-Université Lyon 1, Hôpital Edouard Herriot, 5 place d’Arsonval, 69003 Lyon, France; 3 Immunology Laboratory, Hospices Civils de Lyon, Hôpital Edouard Herriot, 5 place d’Arsonval, 69003 Lyon, France; 4 Anesthesiology and Intensive care department, Hospices Civils de Lyon, Hôpital Edouard Herriot 5 place d’Arsonval, 69003 Lyon, France

**Keywords:** Sepsis, Exhaustion, IL-7, Immunosuppression, CD127, PD-1, T-cell activation

## Abstract

**Background:**

Sepsis is the leading cause of mortality for critically ill patients worldwide. Patients develop T lymphocyte dysfunctions leading to T-cell exhaustion associated with increased risk of death. As interleukin-7 (IL-7) is currently tested in clinical trials to reverse these dysfunctions, it is important to evaluate the expression of its specific CD127 receptor on the T-cell surface of patients with septic shock. Moreover, the CD127^low^PD-1^high^ phenotype has been proposed as a T-cell exhaustion marker in chronic viral infections but has never been evaluated in sepsis. The objective of this study was first to evaluate CD127 and CD127^low^PD-1^high^ phenotype in septic shock in parallel with functional T-cell alterations. Second, we aimed to reproduce septic shock–induced T-cell alterations in an *ex vivo* model.

**Methods:**

CD127 expression was followed at the protein and mRNA levels in patients with septic shock and healthy volunteers. CD127^low^PD-1^high^ phenotype was also evaluated in parallel with T-cell functional alterations after *ex vivo* activation. To reproduce T-cell alterations observed in patients, purified T cells from healthy volunteers were activated *ex vivo* and their phenotype and function were evaluated.

**Results:**

In patients, neither CD127 expression nor its corresponding mRNA transcript level was modified compared with normal values. However, the percentage of CD127^low^PD-1^high^ T cells was increased while T cells also presented functional alterations. CD127^low^PD-1^high^ T cells co-expressed HLA-DR, an activation marker, suggesting a role for T-cell activation in the development of this phenotype. Indeed, T-cell receptor (TCR) activation of normal T lymphocytes *ex vivo* reproduced the increase of CD127^low^PD-1^high^ T cells and functional alterations following a second stimulation, as observed in patients. Finally, in this model, as observed in patients, IL-7 could improve T-cell proliferation.

**Conclusions:**

The proportion of CD127^low^PD-1^high^ T cells in patients was increased compared with healthy volunteers, although no global CD127 regulation was observed. Our results suggest that TCR activation participates in the occurrence of this T-cell population and in the development of T-cell alterations in septic shock. Furthermore, we provide an *ex vivo* model for the investigation of the pathophysiology of sepsis-induced T-cell immunosuppression and the testing of innovative immunostimulant treatments.

**Electronic supplementary material:**

The online version of this article (10.1186/s13054-018-2305-5) contains supplementary material, which is available to authorized users.

## Background

Sepsis has recently been re-defined as a life-threatening organ dysfunction caused by a dysregulated host response to infection [1]. It represents a major health-care challenge with high incidence and mortality [2, 3]. For example, it was recently shown that septic shock (that is, the subset of sepsis associated with acute circulatory failure) is associated with a crude mortality of over 45% [3]. Sepsis also represents a high economic burden, which is due in part to long and costly hospital stays [4].

Patients with sepsis develop severe immune dysfunction affecting innate and adaptive immune responses, whose intensity and duration are associated with increased risk of death and nosocomial infections [5]. Thus, innovative therapies targeting these dysfunctions are being evaluated in sepsis [6]. T lymphocyte dysfunction, or more specifically T-cell exhaustion, occurs during the immunosuppressive phase of sepsis, as previously described in the cancer literature [7, 8]. Indeed, most patients with sepsis are lymphopenic [9], and the remaining T lymphocytes show a poor functional status. This includes decreased proliferation and cytokine production *ex vivo* and increased apoptosis [8, 10, 11] along with an increased expression of co-inhibitory receptors such as PD-1 [12, 13]. Several clinical studies showed that these dysfunctions are associated with increased mortality or secondary infections [8, 12]. Therefore, clinical trials evaluating immuno-adjuvant therapies to target T-cell alterations are ongoing in sepsis.

In particular, preclinical studies showed that IL-7 treatment reduced mortality in murine models of sepsis and improved cell functionality upon *ex vivo* activation of T lymphocytes of patients with septic shock [10, 14, 15]. A recent phase II clinical trial evaluating IL-7 in patients with septic shock showed that IL-7 treatment restored T-cell count in patients with severe lymphopenia in the absence of any severe side effects [16].

IL-7 is a hematopoietic growth factor whose main role is to maintain T-cell homeostasis and favor T-cell functions [17]. IL-7 activity is mediated through its binding to its specific IL-7 receptor (IL-7R). IL-7R is expressed mainly on the T-cell surface and is composed of two chains: an IL-7–specific chain (CD127) and a common receptor γ-chain [18]. In regard to IL-7 functions, IL-7 receptor expression is tightly regulated at both protein and mRNA levels. For example, decreased CD127 expression on T cells has been described in several clinical contexts of T lymphocyte exhaustion, such as human immunodeficiency virus (HIV), hepatitis C virus (HCV) infections, and cancer [19–21]. In sepsis, preliminary data have been generated at the protein level but the corresponding mRNA transcript expression has never been concomitantly studied [10, 12]. In addition, the CD127^low^PD-1^high^ T lymphocyte subset has been proposed as a T-cell exhaustion marker in several clinical contexts [22]. Indeed, CD127^low^PD-1^high^ T cells were identified in chronic viral infections and in cancer. These cells have been shown to display an exhausted functionality with poor cytokine production and proliferation [23–26]. The occurrence of this specific CD127^low^PD-1^high^ phenotype among T cells has never been specifically studied in patients with septic shock.

Thus, in the present study, we aimed to evaluate CD127 expression on T cells at both protein and mRNA levels and the development of CD127^low^PD-1^high^ T cells in parallel with functional alterations of T lymphocytes in patients with septic shock. We also aimed to evaluate the effect of T-cell activation through T-cell receptors (TCRs) on the development of these phenotypic and functional alterations in an *ex vivo* model.

## Material and methods

### Patients and healthy volunteers

This study was conducted in the intensive care unit (ICU) of the Hôpital Edouard Herriot (Hospices Civils de Lyon, Lyon, France). This project was approved by our Institutional Review Board for Ethics (“Comité de Protection des Personnes Sud-Est II”), which waived the need for informed consent, as the study was observational and performed on residual blood, after the completion of routine follow-up (#IRB 11236). This study is registered at the French Ministry of Research and Teaching (#DC-2008-509), at the Commission Nationale de l’Informatique et des Libertés, and on ClinicalTrials.gov (ClinicalTrials.gov Identifier: NCT02803346). Non-opposition to inclusion in the study was registered for each patient. Patients with septic shock were identified in accordance with the diagnostic criteria of the American College of Chest Physicians/Society of Critical Care Medicine and to the new sepsis definition from 2016 [1, 27]. Patients with septic shock were excluded with regard to the following exclusion criteria: age of less than 18, immunosuppressive disease (HIV, cancer, or primary immune deficiency), immunosuppressive or corticoid treatment (dosage of more than 10 mg/day or cumulative dose of more than  700 mg equivalent prednisolone), aplasia as defined by number of circulating neutrophils of less than 500 cells/mm^3^, and extracorporeal circulation during the month prior ICU admission. Nosocomial infections were defined as previously reported [28]. Patients were screened daily during the ICU stay for microbiologically documented pulmonary infection, urinary tract infection, bloodstream infection, and catheter-related infection. Nosocomial infections were defined in accordance with the European Center for Disease Control and Prevention [29]. Expression of HLA-DR on monocytes (mHLA-DR) was measured as previously described and was expressed as the number of anti-HLA-DR antibodies bound per cell (AB/C) [30]. The percentage of CD4^+^CD25^high^CD127^low^ regulatory T (Treg) cells among CD4^+^ T cells was determined as previously described [31]. Peripheral blood samples were collected at three time points after the onset of septic shock: from 24 h to 48 h (D1, *n* = 15), 72 h to 96 h (D3, *n* = 30), and 7 to 8 days (D7, *n* = 10). Peripheral blood from healthy volunteers (HVs) was provided by the “Etablissement Français du Sang”: 34 age-matched HVs for the cohort of patients with septic shock (median age 56 [52–61] years, male 31%) and 13 HVs for the *ex vivo* experiments. According to the standardized procedure for blood donation, written informed consent was obtained from HVs and personal data for blood donors were anonymized at the time of blood donation, prior to blood transfer to our research lab.

### T-cell purification

Human T cells were isolated from HV and septic shock patient samples by antibody-based negative selection and density gradient centrifugation by using a human T-cell enrichment cocktail (Rosette Sep™, StemCell Technologies, Grenoble, France), as described in the instructions of the manufacturer. Quality of T-cell purification was systematically controlled. Purified cells were labeled with a Pacific Blue (PB) anti-CD3 antibody (Ab) (Beckman Coulter, Hialeah, FL, USA) and lithium dodecyl sulfate 751 as a marker of nucleated cells (Molecular Probes, Life Technologies, Saint-Aubin, France). Sample purity was systematically greater than 95%.

### TCR activation and cell culture of T lymphocytes purified from healthy donors

T cells were purified as described above and cultured at 1 × 10^6^ cells per mL in complete culture medium: RPMI 1640 (Eurobio, Les Ulis, France), supplemented with 10% AB human serum (Life Technologies), 2 mM L-Glutamine (Eurobio), 1000 IU/mL penicillin (Eurobio), 1000 μg/mL streptomycin (Eurobio), and 200 μg/mL amphotericin B (Gibco, Thermo Fisher Scientific, Wilmslow, UK). After overnight culture, T cells were seeded in a complete culture medium in 48-well cell culture plates (Corning^®^, Corning, NY, USA) with or without αCD3/28 antibody-coated beads (αCD3/28, T-cell activation/expansion kit, Miltenyi Biotec, Auburn, CA, USA, 1:1 bead-to-cell ratio). T cells cultured with αCD3 antibody-coated beads (αCD3) were used as a control condition, mimicking an incomplete activation and anergy [32]. After 5 days of culture, HLA-DR, CD127, and PD-1 expressions were evaluated by flow cytometry. T-cell functionality (cytokine production and proliferation) was then evaluated, as described below, in response to a secondary stimulation using αCD2/3/28 antibody-coated beads (αCD2/3/28) (T-cell activation/expansion kit, Miltenyi Biotec, 1:1 bead-to-cell ratio) for 3 days. This second stimulation step was intended to mimic experiments performed when evaluating functionality of T lymphocytes from patients with septic shock which are re-stimulated *ex vivo* to induce their effector functions. When IL-7 efficacy in improving T-cell function *ex vivo* was tested, recombinant human IL-7 (100 ng/mL, R&D Systems, Minneapolis, MN, USA) was added simultaneously to αCD2/3/28 for the 3 days of the second activation.

### CD127 mRNA level evaluation

Total RNA was extracted from T cells by using an RNeasy kit (Qiagen, Hilden, Germany). RNA integrity was assessed with an RNA 6000 Nano kit (Agilent Technologies, Santa Clara, CA, USA). Total RNA was reverse-transcribed into complementary DNA (cDNA) by using a SuperScript^®^VILO™ cDNA synthesis kit (Life Technologies). A specific polymerase chain reaction (PCR) assay amplifying the IL7R1 transcript coding for the transmembrane protein (Ensembl release 87) was used with the forward primer CTCTGTCGCTCTGTTGGTC targeting exon 6, the backward primer TCCAGAGTCTTCTTATGATCG targeting exon 7, and the probe CTATCGTATGGCCCAGTCTCC targeting exon 7. Quantitative PCR was conducted on a LightCycler 480 instrument (Roche, Bale, Switzerland) using a LightCycler 480 probes master kit (Roche) in accordance with the instructions of the manufacturer. Thermocycling was performed in a final volume of 20 μL containing 0.5 μM of primers and 0.1 μM of probe, with an initial denaturation step of 10 min at 95 °C, followed by 45 cycles of 10 s denaturation at 95 °C, annealing for 29 s at 68–58 °C, and a 1 s extension period at 72 °C. The crossing point for each sample was calculated by using the second derivative maximum method with the LightCycler software. Standard curves were generated by using five replicates of cDNA standards and were used to perform efficiency-corrected quantification. Results were expressed as absolute concentrations (copy number per microliter) and normalized by the RNA quantity used for reverse transcription.

### Flow cytometry

For septic shock patients and HV cohorts, flow cytometry surface stainings were performed by using 50 μL of whole blood. Erythrocytes were lysed with Versalyse solution (Beckman Coulter). Antibodies were purchased from Beckman Coulter: PB anti-CD4, Krome Orange (KRO) anti-CD8, allophycocyanin (APC) anti-CD127, phycoerythrin-cyanin (PC) 7 anti-CD25, PC7 anti-CD3, phycoerythrin (PE) anti-PD-1, PE anti-CD25, PC5.5 anti-PD-1, PC7 anti-CD127, Alexa Fluor (AF) 700 anti-CD8, and fluorescein isothiocyanate (FITC) anti-CD38 Ab. KRO anti-HLA-DR Ab was purchased from Becton Dickinson (San Jose, CA, USA) and FITC anti-CCR7 Ab from R&D Systems. AF647 anti-FoxP3 Ab was purchased from BioLegend (San Jose, CA, USA). To measure CD127 and PD-1 expressions, corresponding isotypic controls were purchased from Beckman Coulter. All antibodies were used in accordance with concentrations recommended by the manufacturers.

In the *ex vivo* experiments of TCR activation of purified T cells from HVs, 100000 T lymphocytes were stained after 5 days of activation. Antibodies were purchased from Beckman Coulter: PB anti-CD4, FITC anti-CD8, PB anti-HLA-DR, APC anti-CD4, and KRO anti-CD8 Ab. PC7 anti-CD127 and APC anti-PD-1 Ab were purchased from BioLegend.

Flow cytometry assays were performed on a Navios cytometer (Beckman Coulter). Results were expressed as medians of fluorescence intensity (MFIs) for the measurement of CD127 on total CD4^+^ and CD8^+^ T lymphocytes and as percentages of positive cells for all other parameters. Flow cytometry data were analyzed by using Kaluza software (Beckman Coulter).

### Spanning-tree Progression Analysis of Density-normalized Events

T-cell phenotypes of septic shock patients and healthy donors were also evaluated through an unsupervised computational approach using the Spanning-tree Progression Analysis of Density-normalized Events (SPADE) algorithm as described by Qiu *et al*. [33]. Analysis was performed by using Cytobank (Santa Clara, CA, USA).

SPADE analysis was performed on manually gated singulet events from 14 HVs and 17 patients with septic shock. These patients with septic shock were consecutively enrolled during a fixed period of time. Nine of them were also included in the analysis of cytokine production and IL-7 receptor and PD-1 expressions. We verified that the clinical characteristics of this subgroup of patients did not differ from those of the entire cohort. Raw median intensity values were transformed to a hyperbolic arcsine (arcsinh) scale with a cofactor of 5. The target numbers of nodes were adjusted to 4 for CD4^+^ T cells and 3 for CD8^+^ T cells. Clustering channels included all CD127, CD38, HLA-DR, PD-1, FoxP3, and CD25 parameters. Percentage downsampling was 10%. Intensity and cellular abundance of each node from each individual were exported for further analysis. Relative expressions of each marker in each node were then compared by the MFI of cells included in the node. Finally, the percentages of cells in each node were compared between patients with septic shock and HVs.

### Intracellular cytokine stainings

For patients with septic shock and HVs, intracellular cytokine contents were measured from a 50 μL whole blood sample after 3 h of incubation at 37 °C with pre-coated tubes containing phorbol 12-myristate 13-acetate (PMA), ionomycin, and brefeldin A (Beckman Coulter). The mitogen PMA is a classic stimulant for intracellular cytokine production experiments [34], which we had previously evaluated on samples from patients with septic shock [35]. Intracellular staining was then performed by using a PerFix-nc no centrifuge assay kit (Beckman Coulter) and tubes containing dry AF750 anti-CD3, PB anti-CD4, AF700 anti-CD8, FITC anti-interferon gamma (anti-IFNγ), PE anti-tumor necrosis factor alpha (anti-TNFα), and PC7 anti-IL-2 Ab (DuraClone IF T Activation Tube, Beckman Coulter). Gating was performed as previously described [35].

In the *ex vivo* experiments evaluating TCR activation of purified T cells from HVs, T lymphocytes were incubated with brefeldin A (Sigma-Aldrich, St. Louis, MO, USA) at a final concentration of 10 μg/mL during the last 4 h of the secondary stimulation step at 37 °C in 5% CO_2_. T cells were then stained for surface markers: PC7 anti-CD4 and KRO anti-CD8 Ab (Beckman Coulter). Samples were then fixed using Iotest 3 fixing solution (Beckman Coulter) and permeabilized using BD perm/wash buffer (Becton Dickinson) in accordance with the instructions of the manufacturer. Samples were then stained for intracellular cytokines: FITC anti-IFNγ and PE anti-IL-2 Ab (Beckman Coulter) and APC anti-TNFα Ab (BioLegend). Results were expressed as percentages of positive cells for each cytokine or for the three cytokines among CD4^+^ and CD8^+^ T cells.

### Proliferation assay

In the *ex vivo* experiments of TCR activation of T cells purified from HVs, T-cell proliferation was evaluated after 3 days of re-activation using αCD2/3/28 using a Click-it AF488 EdU Flow Cytometry assay kit (Thermo Fisher Scientific) in accordance with the instructions of the manufacturer and the protocol used in our lab [36]. Cells were additionally stained with APC anti-CD4 and APC-AF750 anti-CD8 Ab from Beckman Coulter. Results were expressed as percentages of EdU-positive cells (that is, proliferating cells) among CD4^+^ or CD8^+^ T cells.

### Statistical analysis

Results are presented as Tukey boxplots. Bottom and top of the box represent the first and third quartiles, respectively. The horizontal bar within the box represents the median value. Lower and higher extremities of the whiskers respectively represent the lowest datum still within 1.5 interquartile range of the lower quartile and the highest datum still within 1.5 interquartile range of the upper quartile. Mann–Whitney unpaired tests were used to assess variations between patients with septic shock and HVs, and Mann–Whitney paired tests were used to assess variations between *ex vivo* non-stimulated and activated T cells purified from HVs. Statistical analyses were performed by using R statistics software (R version 3.2.4). *P* values lower than 0.05 were considered statistically significant.

## Results

### Cohort of patients with septic shock

Thirty-one patients with septic shock were included in the study. Their clinical and biological data are presented in Table. 1. Twenty-three (73%) were still alive after 28 days. The median Sequential Organ Failure Assessment (SOFA) score at admission was 8 and the median Simplified Acute Physiology Score II (SAPS II) score was 60, illustrating the severity of this cohort. The median lymphocyte count at D3 was 1.05 G/L, which is below normal value [37]. The median HLA-DR expression on monocytes was 7748 AB/C, characteristic of sepsis-induced immunosuppression [38]. Thirty-four age-matched healthy donors were also included (median age 56 [52–61] years, male 31%).

**Table 1:** Clinical characteristics of patients with septic shock

Parameters	Patients with septic shock (*n* = 31)
Sex, male	19 (62%)
Age, years	72 [63.5–76.5]
SAPS II	60 [49.5–75.5]
SOFA (*n* = 29)	8 [8–11]
Charlson co-morbidity score	
0	4 (13%)
1	13 (42%)
>1	14 (45%)
Initial infection	
Abdominal infection	14 (45%)
Urinary infection	4 (13%)
Pneumopathy	1 (3%)
Other	12 (39%)
Type of admission	
Medical	19 (61.5%)
Emergency surgery	11 (35.5%)
Elective surgery	1 (3%)
Microbiological documentation	
Gram-negative	8 (26%)
Gram-positive	8 (26%)
Other	1 (3%)
Non-documented	14 (45%)
Mortality at day 28	8 (27%)
Nosocomial infections	1 (3%)
ICU length of stay, days	6 [5–13]
Lactate at admission, mmol/L (*n* = 28)	3.35 [2.5–5.83]
Lymphocytes at day 3, G/L (n = 21)	1.05 [0.55–1.45]
mHLA-DR at day 3, AB/C	7748 [3358–15,200]
Percentage of Treg cells among CD4^+^ cells at day 3 (n = 20)	5.0 [3.0–6.8]

For clinical parameters, categorical data are presented as numbers of cases and percentages of the total population in brackets. Continuous data and biological parameters are presented as medians and interquartile ranges [Q1–Q3]. SAPS II (Simplified Acute Physiology Score II) was calculated on admission. SOFA (Sequential Organ Failure Assessment) score was measured after 24 h of intensive care unit (ICU) stay. mHLA-DR (AB/C): number of anti-HLA-DR antibodies bound per monocyte. Regulatory T (Treg): CD4^+^CD25^high^CD127^low^ Treg cells

**Fig. 1:**
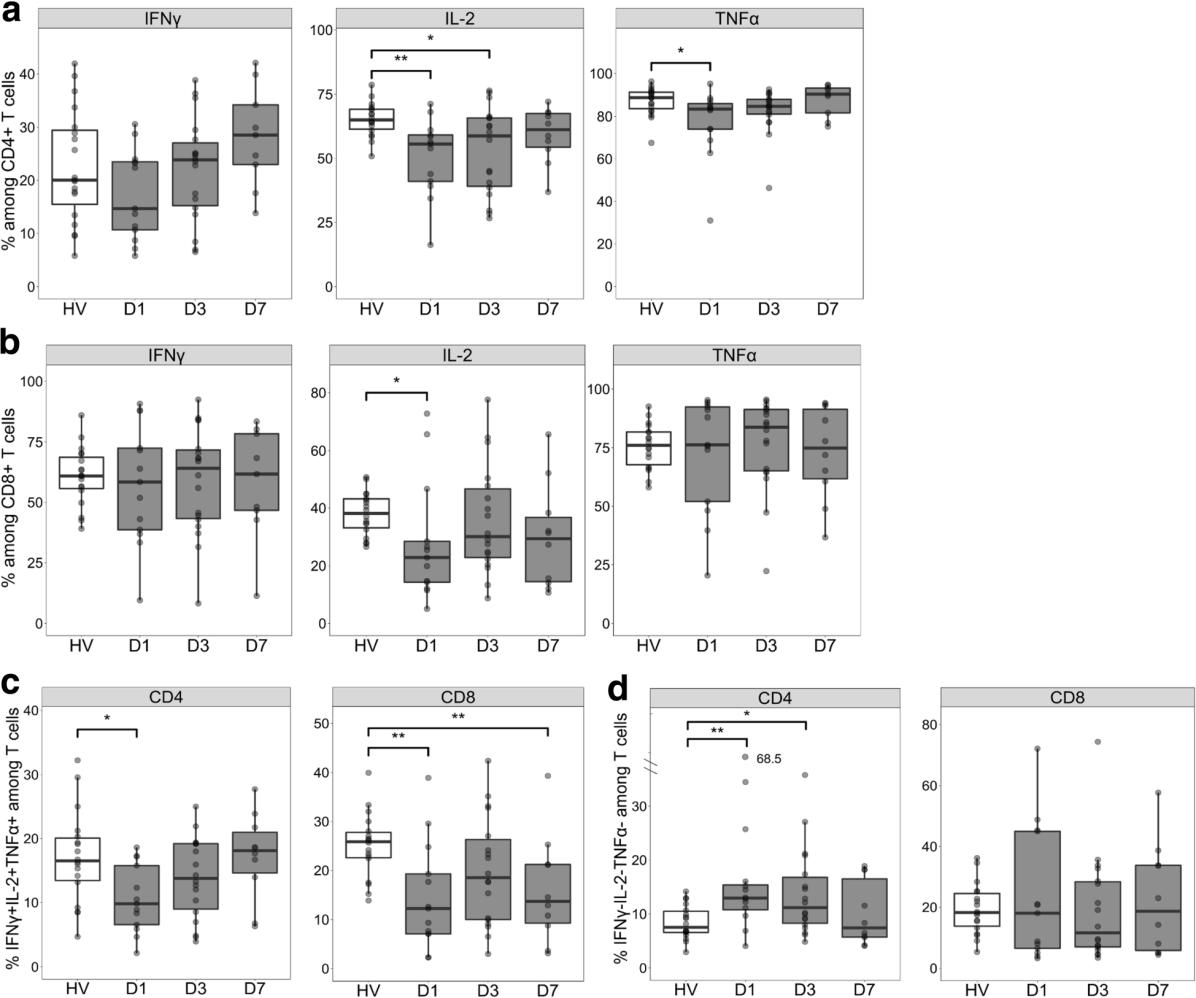
Cytokine production in T cells of patients with septic shock compared with those of healthy volunteers (HVs). Intracellular interleukin-2 (IL-2), tumor necrosis factor alpha (TNFα), and interferon gamma (IFNγ) staining was performed after 3 hours of T-cell activation using phorbol 12-myristate 13-acetate (PMA)/ionomycin in whole blood samples from patients with septic shock at day 1 (D1, *n* = 14), day 3 (D3, *n* = 18), and day 7 (D7, *n* = 10) after the onset of shock in comparison with HVs (n = 18). The percentages of cells producing each cytokine (IFNγ: left panel, IL-2: middle panel, and TNFα: right panel) are represented among CD4^+^ (**a**) and CD8^+^ (**b**) T cells. The percentages of polyfunctional cells simultaneously producing the three cytokines (**c**) or none of the three cytokines (**d**) are represented among CD4^+^ (left panel) and CD8^+^ (right panel) T cells. Data are presented as Tukey boxplots. Mann–Whitney tests were used to compare values between patients with septic shock and HVs, *P <0.05, **P <0.01

In order to evaluate the occurrence of sepsis-induced T-cell functional alterations in this cohort, we measured intracellular IL-2, TNFα, and IFNγ productions after *ex vivo* activation of T lymphocytes from patients and HVs. As expected, cytokine production was partially decreased in T cells of patients compared with those of HVs. We observed that the percentage of IL-2–producing cells was significantly decreased among CD4^+^ T cells at D1 and D3 and among CD8^+^ T cells at D1 in patients with septic shock in comparison with HVs (Fig. 1a and b). In addition, the percentage of TNFα–producing cells was decreased among CD4^+^ T cells at D1 but not in CD8^+^ T cells after septic shock (Fig. 1a and b). When evaluating the proportion of polyfunctional T cells, we showed that the proportion of triple-positive CD4^+^ and CD8^+^ T cells (that is, IL-2^+^TNFα^+^IFNγ^+^–producing cells) was greatly reduced at D1 in patients with septic shock compared with controls (Fig. 1c). This decrease was also observed in CD8^+^ T cells and persisted until D7 after the onset of shock. In comparison, the proportion of triple-negative CD4^+^ T cells (that is, cells producing none of the three cytokines) was increased at D1 and D3, but no strong modifications were observed in CD8^+^ T cells (Fig. 1d)*.*

### Increased proportion of CD127^low^PD-1^high^ T cells after septic shock

**Fig. 2:**
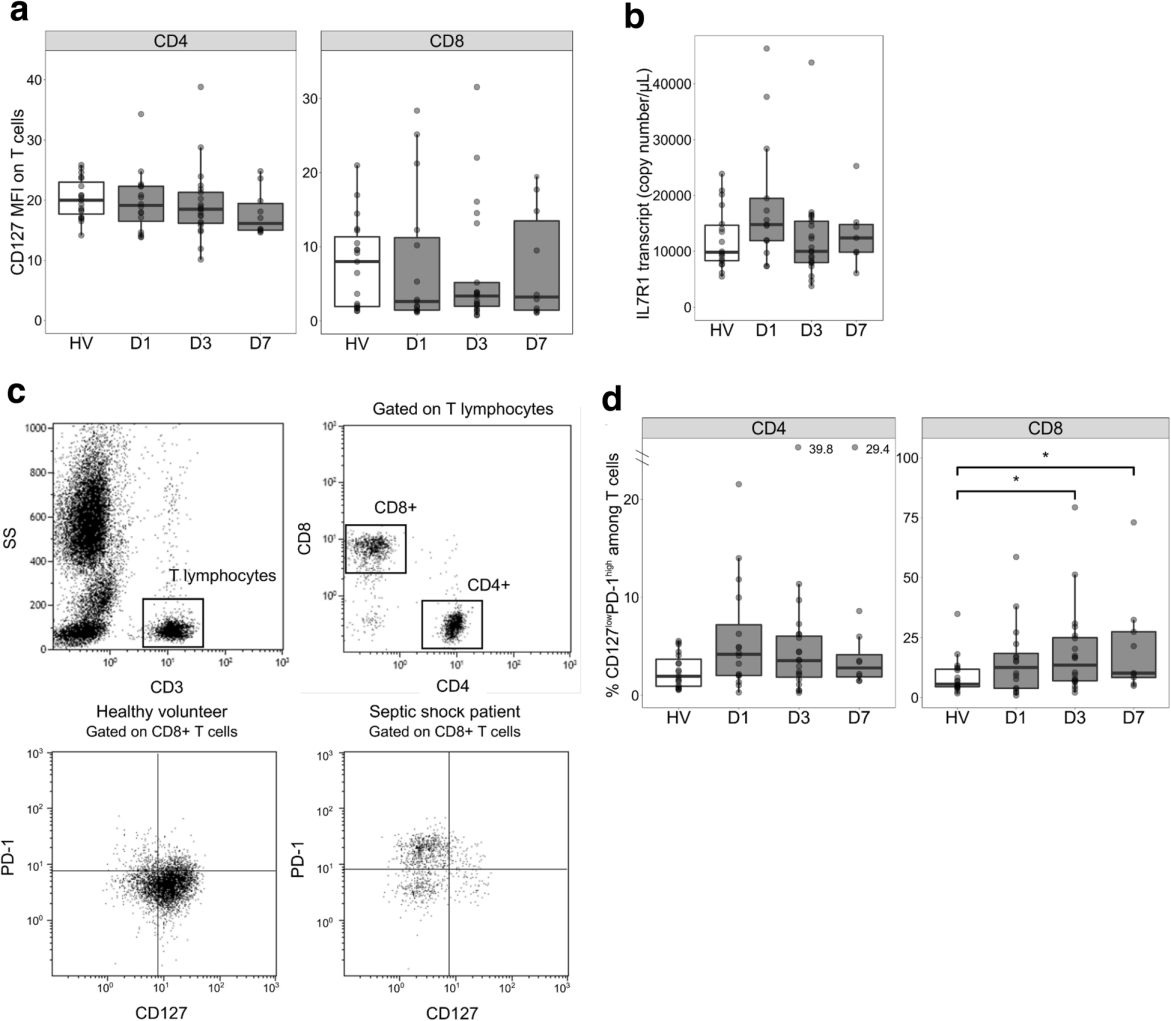
CD127 expression and CD127^low^PD-1^high^ T cells in patients with septic shock and healthy volunteers (HVs). **a** Cell surface expression of CD127 was measured by flow cytometry on septic shock patients’ CD4^+^ (left panel) and CD8^+^ (right panel) T cells at day 1 (D1, *n* = 15), day 3 (D3, *n* = 21), and day 7 (D7, n = 10) after the onset of shock in comparison with HVs (*n* = 20). Results are expressed as median of fluorescence intensity (MFI) on selected CD4^+^ and CD8^+^ T cells. **b** Expression of IL7R1 mRNA transcript, coding for cell surface CD127, was measured by reverse transcription quantitative polymerase chain reaction using RNA extracted from purified T cells at D1 (*n* = 13), D3 (n = 20), and D7 (*n* = 7) for patients with septic shock in comparison with HVs (n = 18). **c** Gating strategy for the determination of the percentage of CD127^low^PD-1^high^ T cells. Sequential gating was used to select first the CD3^+^ T-cell population among all leukocytes and then CD3^+^ CD4^+^ and CD3^+^ CD8^+^ T-cell subsets among CD3^+^ cells. PD-1 and CD127 expression thresholds were defined using isotype controls. One example of a CD127 and PD-1 staining among CD8^+^ T cells in one HV and one septic shock patient are represented. **d** The percentage of CD127^low^PD-1^high^ among CD4^+^ (left panel) and CD8^+^ (right panel) T cells at day 1 (D1, n = 15), day 3 (D3, n = 21), and day 7 (D7, n = 10) after the onset of shock in comparison with HVs (n = 20) is represented. Data are presented as Tukey boxplots. Mann–Whitney tests were used to compare values between patients with septic shock and HV, *P <0.05.

We evaluated CD127 surface expression on circulating CD4^+^ and CD8^+^ T cells from patients and HVs (Fig. 2a). CD127 expression on the membrane of CD4^+^ and CD8^+^ T cells did not differ between patients with septic shock and HVs at any time point. We observed only a slight but not significant decrease on CD8^+^ T cells at D3. The expression of cell surface CD127 corresponding transcript IL-7R1 was similar between purified T cells from patients with septic shock and those of HVs (Fig. 2b). This result is in agreement with the absence of regulation of CD127 expression on T lymphocyte surface. Thus, membrane IL-7R expression is not strongly deregulated at both protein and transcript levels in T cells of patients with septic shock compared with those of HVs.

We then investigated whether CD127 expression might be deregulated on some specific T-cell subpopulations. In particular, the CD127^low^PD-1^high^ phenotype has been proposed as a marker of T-cell exhaustion in several clinical contexts [22, 39]. We therefore measured PD-1 and CD127 expressions on circulating T cells from patients with septic shock and HVs. In accordance with the literature [13], we observed that PD-1 was significantly overexpressed on CD4^+^ T cells at D3 in patients with septic shock compared with HVs (Additional file 1: Figure S1). Meanwhile, the percentage of CD127^low^PD-1^high^ CD8^+^ T cells was significantly increased in patients with septic shock at D3 and D7 compared with HVs. This percentage also tended to be higher in CD4^+^ T cells from patients at D3 and D7 compared with HVs (Fig. 2c and d).

### CD127^low^PD-1^high^ T lymphocytes expressed HLA-DR, a late activation marker on T cells

In addition to being a marker of T-cell exhaustion, PD-1 expression is known to be increased on T cells after activation [40]. Thus, we next evaluated whether this CD127^low^PD-1^high^ phenotype was associated with increased expression of activation markers on T lymphocytes after septic shock. For this, we used an unsupervised computational approach using a SPADE algorithm to cluster cells in nodes according to their expressions of activation/exhaustion markers measured by flow cytometry. Indeed, whereas traditional methods for flow cytometry data analysis are based on the selection of subsets of cells in a process called “gating” (a gate being a region, defined in a biaxial plot of two measurements, used to select cells with a desired phenotype for downstream analysis), SPADE clusters cells into populations (or nodes) on the basis of their similarities in phenotypes for all the markers included in the staining without any *a priori* considerations on the expected cell subpopulations or marker expressions on these cell subtypes.

**Fig. 3:**
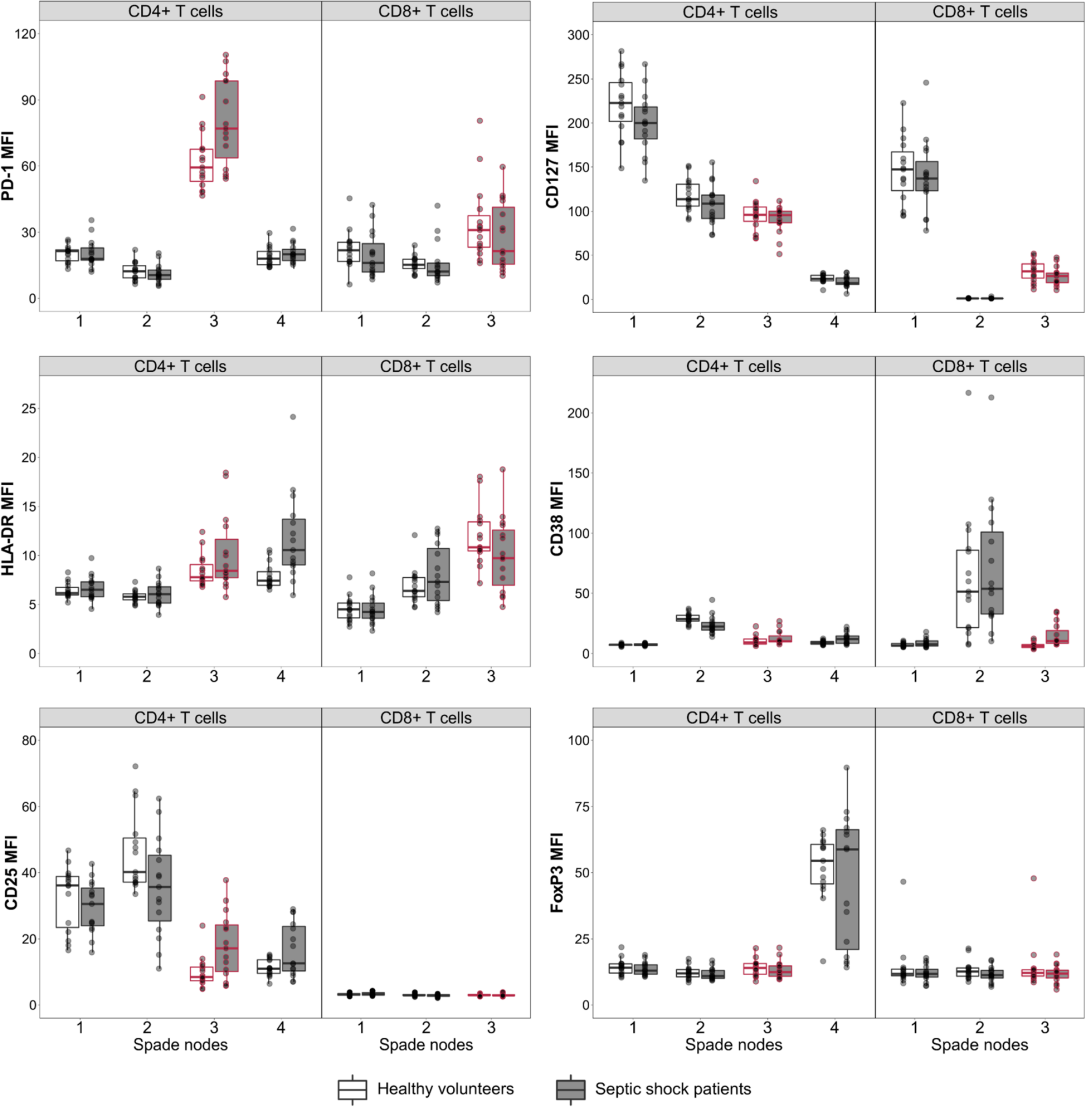
SPADE (Spanning-tree Progression Analysis of Density-normalized Events) analysis. T-cell phenotype was evaluated by using a SPADE algorithm based on the expression of different markers measured by flow cytometry on whole blood samples from patients with septic shock at day 3 after the onset of shock (D3, *n* = 17) and in healthy volunteers (HVs) (n = 14). CD4^+^ and CD8^+^ T cells from patients and donors were clustered in nodes on the basis of their similarities of expressions of CD127, CD38, HLA-DR, PD-1, FoxP3, and CD25. One tree containing four nodes was built for CD4^+^ T lymphocytes and one tree containing three nodes for CD8^+^ T cells. The medians of fluorescence intensity (MFIs) of the different markers for each node are represented on CD4^+^ and CD8^+^ T cells in patients and in HVs. Data are presented as Tukey boxplots. Node 4 of the CD4^+^ T cells, with a CD127^low^CD25^high^FoxP3^high^ phenotype, corresponds to regulatory T cells. Node 3 corresponding to CD127^low^PD-1^high^ cells has been highlighted in both CD4^+^ and CD8^+^ T cells

Thus, we performed an eight-color staining of CD4^+^ and CD8^+^ lymphocytes with CD127, CD38, HLA-DR, PD-1, FoxP3, and CD25 markers in 14 healthy donors and 17 patients with septic shock and we parametered SPADE software to cluster these cells in four subpopulations (or nodes) for CD4^+^ and three subpopulations for CD8^+^ T cells with similar phenotypes. Cell surface marker expressions in each node in patients with septic shock and HVs are presented in Fig. 3. The proportions of each node between patients and HVs are presented in Additional file 2: Figure S2.

First, it was observed that, among the different nodes, PD-1 was highly expressed only on node number 3 in both CD4^+^ and CD8^+^ T cells (Fig. 3). Moreover, node number 3 in CD4^+^ and CD8^+^ T cells displayed a CD127^low^ phenotype, thus corresponding to CD127^low^PD-1^high^ T cells previously mentioned. This confirms that the increased PD-1 expression on CD4^+^ and CD8^+^ T cells from patients and controls was solely observed on CD127^low^ T cells both in CD4^+^ and CD8^+^ cells. In addition, the identification of this specific cell subset using an unsupervised analysis reinforces the results obtained using a classic flow cytometry analysis. Interestingly, in this subgroup of patients, the proportion of node 3 in CD4^+^ T cells was higher in patients with septic shock than in HVs (Additional file 2: Figure S2).

In addition, we showed that these CD127^low^PD-1^high^ CD4^+^ T cells were a different cell population than regulatory CD4^+^ Foxp3^+^ T cells as a significant Foxp3 expression was observed in node 4 among the CD4^+^ T cells but not in any other node.

Finally, this CD127^low^PD-1^high^ phenotype was associated with a high expression of HLA-DR, a late activation marker on T lymphocytes, but not of CD38 and CD25 (earlier activation markers) on both CD4^+^ and CD8^+^ T cells (Fig. 3) [41]. We therefore show that the proportion of CD127^low^PD-1^high^ T lymphocytes is increased after septic shock, especially in CD8^+^ T cells, and that these cells co-express a high level of HLA-DR, a late activation marker, concomitantly with circulating T-cell functional alterations.

### Sepsis-induced CD127^low^PD-1^high^ phenotype and functional T-cell alterations are reproduced *ex vivo* after TCR activation

As CD127 and PD-1 are known to be regulated on T cells following activation, and in light of the high HLA-DR expression on CD127^low^PD-1^high^ T cells, we hypothesized that T-cell activation through TCR may play a role in the occurrence of this specific phenotype in T cells of patients with septic shock. We also further questioned whether such initial activation could lead to functional alterations to a second TCR challenge as observed in septic shock. To test this hypothesis, we evaluated phenotype and functions of purified T cells from HVs following *ex vivo* TCR activation.

**Fig. 4:**
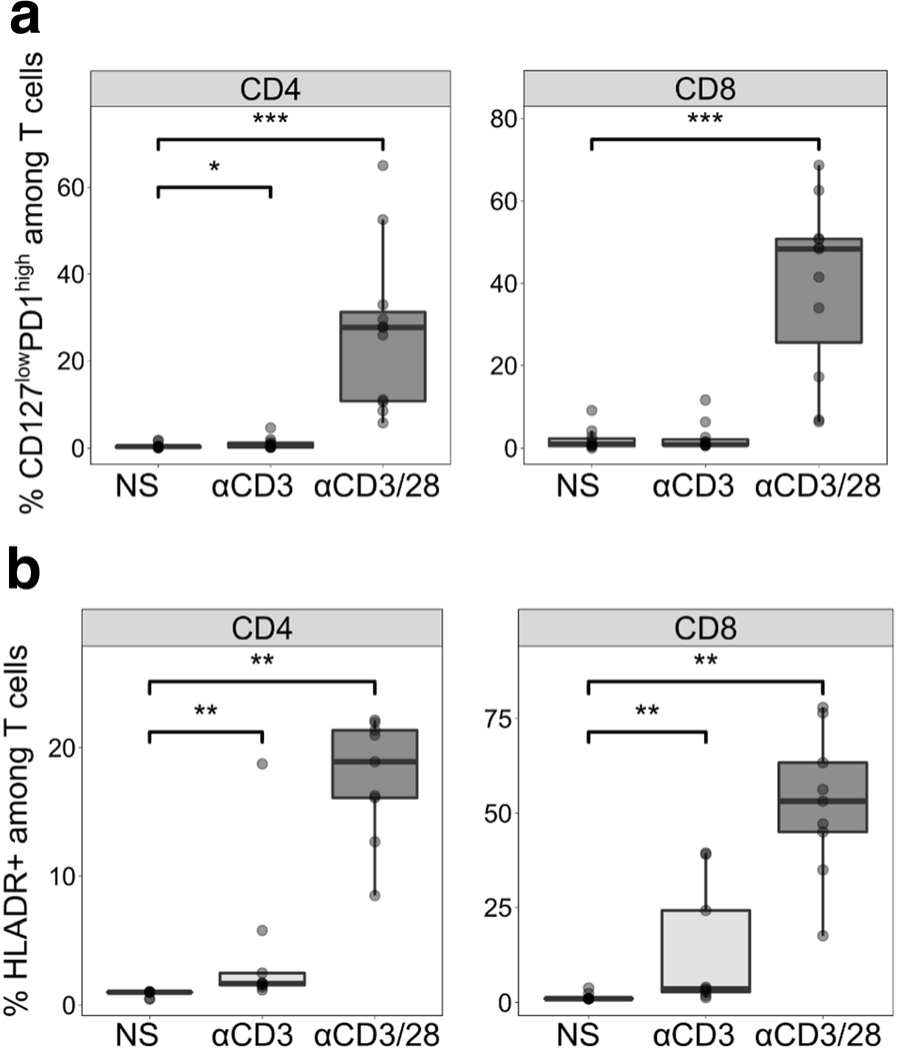
Occurrence of CD127^low^PD-1^high^ T cells in an *ex vivo* model of T-cell receptor activation of purified T cells from healthy volunteers (*n* = 9). Purified T cells were activated with anti-CD3/28 antibody-coated beads (αCD3/28, 1:1 bead-to-cell ratio) or anti-CD3 antibody-coated beads (αCD3, 1:1 bead-to-cell ratio) or not stimulated (NS) during 5 days. The percentage of CD127^low^PD-1^high^ (**a**) as well as the percentage of HLA-DR (**b**) positive cells were measured by flow cytometry among CD4^+^ (left panel) and CD8^+^ (right panel) T cells. Data are presented as Tukey boxplots. Mann–Whitney paired tests were used to compare values between non-stimulated and activated conditions, *P <0.05, **P <0.01, ***P <0.001

Purified T cells from HVs were cultured for 5 days with αCD3/28, mimicking complete TCR activation. CD3 stimulation alone, mimicking incomplete TCR activation and anergy [32], and non-stimulated T cells were used as controls. When evaluating the proportion of CD127^low^PD-1^high^ T cells, we observed that this population was strongly induced in fully activated CD4^+^ and CD8^+^ T cells but not in T cells stimulated through CD3 alone or cells left untreated (Fig. 4a). Moreover, CD127^low^PD-1^high^ T cells represented a large proportion of T cells in this experimental condition: around 30% of CD4^+^ and 50% of CD8^+^ T cells. We also observed that CD3/28 activated T cells expressed high levels of HLA-DR in comparison with non-stimulated T cells or cells stimulated through CD3 alone (Fig. 4b). As a result, we show that a complete *ex vivo* TCR activation of purified T cells from healthy donors led to the induction of CD127^low^PD-1^high^ T cells and HLA-DR expression, as observed in patients with septic shock.

**Fig. 5:**
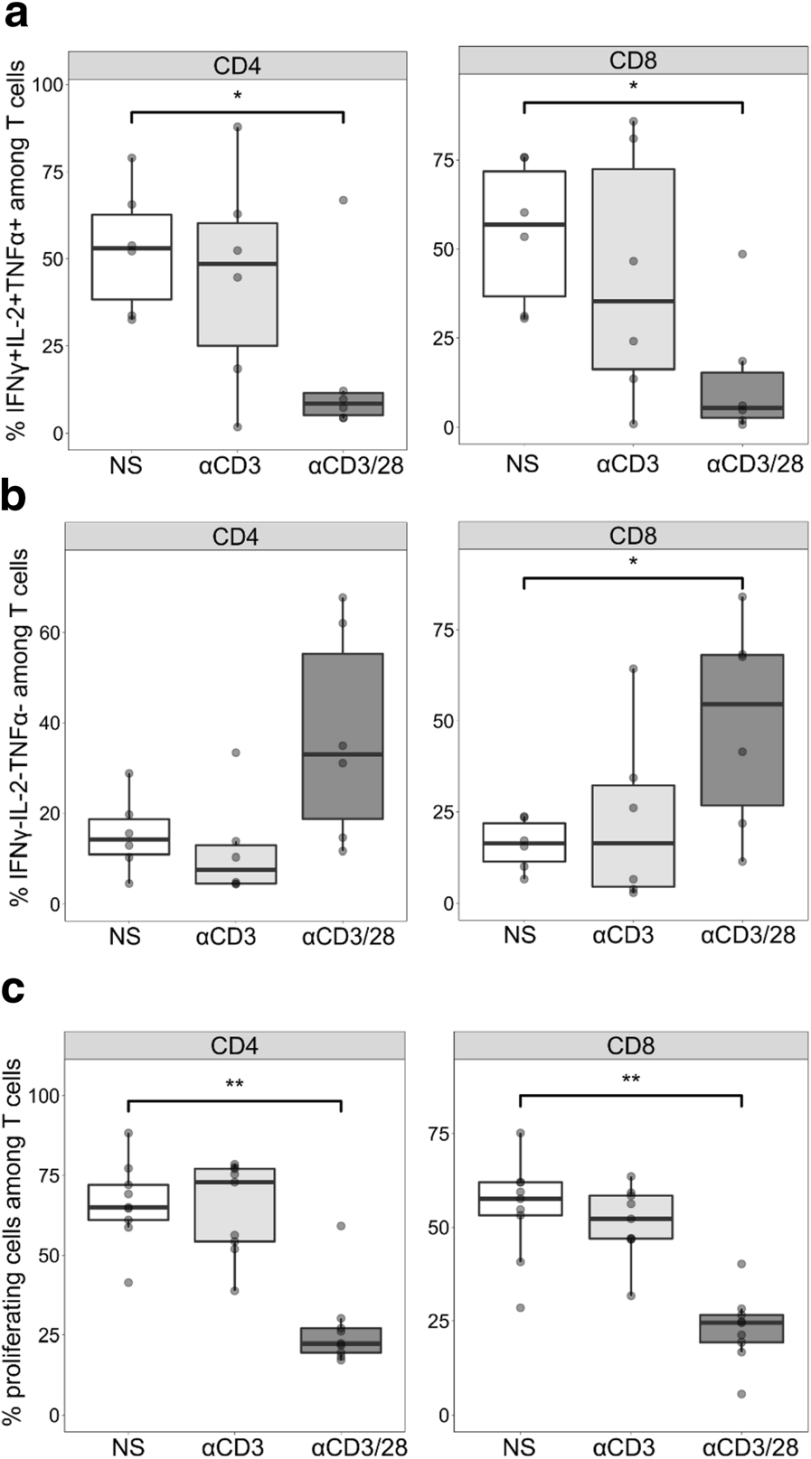
Altered T-cell functionality in an *ex vivo* model of T-cell receptor activation of purified T cells from healthy volunteers (n = 9). Purified T cells were activated with anti-CD3/28 antibody-coated beads (αCD3/28, 1:1 bead-to-cell ratio) or anti-CD3 antibody-coated beads (αCD3, 1:1 bead-to-cell ratio) or not stimulated (NS) for 5 days. T cells were then activated a second time with anti-CD2/3/28 antibody-coated beads (αCD2/3/28, 1:1 bead-to-cell ratio) for 3 days. Intracellular interleukin 2 (IL-2), tumor necrosis factor alpha (TNFα), and interferon gamma (IFNγ) staining was performed. The percentages of cells producing the three cytokines simultaneously (**a**) or none of the three cytokines (**b**) are represented for CD4^+^ (left panel) and CD8^+^ (right panel) T cells. **c** The percentages of proliferating cells are represented for CD4^+^ (left panel) and CD8^+^ (right panel) T cells. Data are presented as Tukey boxplots. Mann–Whitney paired tests were used to compare values between non-stimulated and stimulated conditions, *P <0.05, **P <0.01

We then tested whether such activated T lymphocytes also presented with functional alterations to a second stimulation *ex vivo* as observed in patients. T cells activated *ex vivo* were subsequently activated with αCD2/3/28 and their cytokine production and proliferation were measured (Fig. 5). As observed in patients with septic shock, cytokine production was altered. The percentages of triple-positive IL-2^+^TNFα^+^IFNγ^+^ T cells were markedly decreased in the αCD3/28 activated condition compared with the non-stimulated condition in both CD4^+^ and CD8^+^ (Fig. 5a) T cells. The percentages of triple-negative IL-2^−^IFNγ^−^TNFα^−^ T lymphocytes were also markedly increased in CD8^+^ T cells and with a strong trend in CD4^+^ T cells (Fig. 5b). Upon incomplete TCR activation with CD3 stimulation alone, the percentages of triple-positive cells among CD4^+^ and CD8^+^ T cells were slightly decreased whereas the percentages of triple-negative cells were stable among CD4^+^ and slightly increased among CD8^+^ T cells in comparison with non-stimulated T cells. In regard to T lymphocyte proliferation, T-cell activation through αCD3/28 stimulation induced a significant decrease in both CD4^+^ and CD8^+^ proliferating T cells following a second activation (Fig. 5c). Incomplete activation with αCD3 induced no major difference in the proliferation for both CD4^+^ and CD8^+^ T cells.

### IL-7 partially restored T-cell functionality in the *ex vivo* model of TCR activation

As IL-7 has been shown to efficiently restore sepsis-induced T-cell proliferation in *ex vivo* T cells of patients with sepsis and in murine models of sepsis [10, 14], we tested IL-7 efficacy in restoring a functional status in the current model of TCR activation of T cells from HVs. After the activation phase with αCD3/28, T cells purified from HVs were re-stimulated *ex vivo* in the presence or absence of IL-7 and proliferation was measured.

**Fig. 6:**
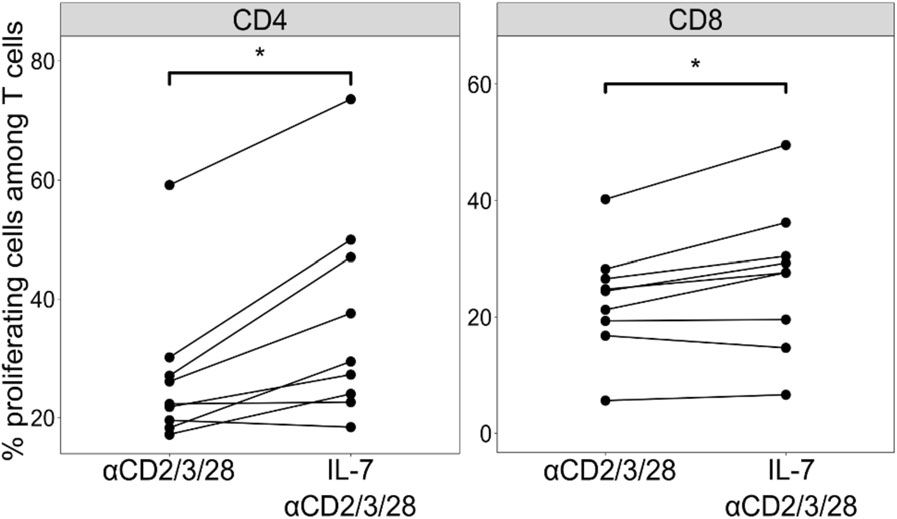
Effect of interleukin-7 (IL-7) treatment on T-cell proliferation in an *ex vivo* model of T-cell receptor activation of purified T cells from healthy volunteers (n = 9). T-cell proliferation was evaluated on αCD3/28 pre-activated T cells after 3 days of activation using αCD2/3/28 antibody-coated beads in the presence and absence of IL-7. Results are presented for CD4^+^ (left panel) and CD8^+^ (right panel) T cells. Mann–Whitney paired tests were used to compare values with IL-7 with those without, *P <0.05

We observed that the percentages of proliferating T cells were significantly increased in the presence of IL-7 for both CD4^+^ and CD8^+^ T cells compared with T cells stimulated in the absence of this cytokine (Fig. 6). This signified that, in this model of TCR activation as observed in T cells from patients with septic shock, IL-7 could improve T-cell proliferation. This final result further supports the capacity of our model to reproduce e*x vivo* sepsis-induced intrinsic T-cell alterations.

Overall, the present model of TCR activation of T cells from healthy donors reproduced *ex vivo* phenotypic and functional alterations of T cells, as described in patients with septic shock. In addition, as observed in patients, decreased proliferation could be improved by IL-7 in this model.

## Discussion

In the present study, we showed that, despite the absence of a global regulation of CD127 expression on CD4^+^ and CD8^+^ T cells, the proportion of CD127^low^PD-1^high^ cells among T lymphocytes was higher in patients than in controls, as were T lymphocyte functional alterations. Interestingly, *ex vivo* TCR activation of purified T cells from HVs also produced the increased proportion of CD127^low^PD-1^high^ T cells and their functional alterations. In this model, as observed in patients [10], IL-7 could improve T-cell proliferation. This suggests that initial T-cell activation through TCR may participate in sepsis-induced T-cell immunosuppression. Moreover, we describe an *ex vivo* model to further investigate the pathophysiology of sepsis-induced T-cell immunosuppression as well as to test innovative immunostimulant treatments *ex vivo*.

A recent reassessment in sepsis pathophysiology has led to a significant gain of interest in the central role of acquired immunosuppression in this clinical context [1]. Sepsis, and in particular septic shock, is associated with the development of immune dysfunction affecting both innate and adaptive immune responses [6]. Importantly, intensity and duration of sepsis-induced immune alterations are associated with increased risk of secondary infections and mortality, therefore identifying immune alterations as a novel therapeutic target in sepsis [8, 42]. T lymphocyte response is highly dysfunctional in patients, and a state of T-cell exhaustion rapidly develops after sepsis [8, 11]. Indeed, lymphocyte count, phenotype, and effector functions are markedly altered in patients. The first phase II clinical trial of IL-7 indicated the capacity of the IL-7 treatment in restoring T-cell count in septic shock patients with severe lymphopenia [16]. Therefore, it appears worthwhile to further delineate the expression of its specific cell surface receptor in patients with septic shock.

Overall, we did not observe any major regulation of membrane IL-7R expression on circulating T cells, at either the protein or the transcriptional level. This result is in contrast with CD127 regulation on circulating T cells described in cancer and chronically infected patients. Indeed, a decreased CD127 expression on circulating T cells has been described in patients with HIV, HCV, or cancer [19–21]. Thus, although it has been suggested that sepsis-induced immune alterations present some similarity with T-cell exhaustion observed in cancer or chronic infections [43], CD127 regulation on circulating T cells is different in these clinical contexts.

Nonetheless, the absence of CD127 regulation on T cells after septic shock is consistent with results from Boomer *et al.*, who also observed a similar CD127 surface expression in T cells of patients with sepsis 24 h after the onset of shock compared with HVs [12]. The overall absence of membrane IL-7R regulation in patients strengthens the use of IL-7 in septic shock and is consistent with preliminary data in preclinical models of sepsis which showed the efficacy of this molecule in restoring sepsis-induced T-cell alterations [10, 14, 15, 44]. Moreover, CD127 surface expression was reduced in T cells of patients with septic shock following IL-7 treatment and therefore could be evaluated as a biomarker of the efficiency of IL-7 therapy [16].

This absence of global regulation of CD127 on total CD4^+^ and CD8^+^ T cells in patients with septic shock does not preclude the regulation of this molecule on any specific T-cell subpopulation. In particular, the CD127^low^PD-1^high^ phenotype has been described in clinical contexts characterized by T-cell exhaustion, such as cancer and HIV and HCV infections. For example, CD127^low^PD-1^high^ T cells were identified in tumor-infiltrating T lymphocytes from patients with ovarian cancer and in liver-infiltrating T lymphocytes from patients with HCV [23, 24]. Moreover, these CD127^low^PD-1^high^ HCV-specific T cells showed an altered proliferation upon *ex vivo* stimulation [25]. Upon *ex vivo* activation, CD8^+^ CD127^low^PD-1^high^ HIV-specific T cells presented an altered production of IL-2 and TNFα [26]. The culmination of these results led to the description of the CD127^low^PD-1^high^ phenotype as an exhaustion marker [22]. Interestingly, we describe here for the first time a higher proportion of CD127^low^PD-1^high^ T cells in patients with septic shock compared with HVs in both CD4^+^ and CD8^+^ T cells. In addition, we show that these cells represent a cell population distinct from CD25^high^CD127^low^ CD4^+^ T cells or Treg cells. We previously reported that patients with septic shock presented a high proportion of Treg cells, which have been shown to participate to sepsis-induced immunosuppression [31]. In the present study, the unsupervised SPADE clustering of T lymphocytes showed that the subpopulation of CD127^low^PD-1^high^ T cells was clustered in a node different from that of Foxp3^+^ CD4^+^ T cells. In addition, this analysis showed that Treg cells did not overexpress PD-1. Finally, we showed that when measured in all patients, the percentage of Treg cells among CD4^+^ was not correlated with the proportion of CD127^low^PD-1^high^ CD4^+^ T cells (Spearman correlation coefficient was 0.23, data not shown). This phenotype occurred in parallel with T-cell functional alterations such as decreased cytokine production *ex vivo*, illustrating the development of T-cell exhaustion in patients with septic shock, as observed in chronic viral infections. This reinforces the similarities between T-cell exhaustion in chronic viral infection and in sepsis. Furthermore, as proposed in other clinical contexts, this suggests that the proportion of CD127^low^PD-1^high^ T cells could be evaluated as a specific biomarker of T-cell dysfunctions in septic shock. This also suggests that similar mechanisms may lead to increased CD127^low^PD-1^high^ T cells and T-cell functional alterations in chronic viral infections and in sepsis.

In particular, we showed that these cells also expressed the late activation marker HLA-DR. A similar phenotype CD127^low^PD-1^high^HLA-DR^high^ was already described in tumor-infiltrating T lymphocytes from metastatic melanoma patients and was associated with an exhausted CD8^+^ T-cell functionality [45]. Although chronic antigen stimulation has been proposed to participate in T-cell exhaustion in chronic viral infection and cancer, the mechanisms leading to T-cell alterations in sepsis are not fully understood [7]. The co-expression of a late activation marker in parallel with a characteristic phenotype of T-cell exhaustion suggests a potential role for T-cell activation in the development of T-cell alterations following septic shock. Similarly, it has been shown that T cells from patients with sepsis present an activated phenotype [46, 47]. Furthermore, this potential role for T-cell activation is supported by *ex vivo* data. Indeed, we showed that after *ex vivo* TCR activation, and as observed in patients with septic shock, the proportion of CD127^low^PD-1^high^ T cells was increased compared with un-stimulated cells or cells stimulated through CD3 alone. Additionally, these cells were unresponsive to a second TCR activation showing decreased percentages of polyfunctional CD4^+^ and CD8^+^ T cells and decreased cell proliferation. Finally, the present model of TCR activation of purified T cells from HVs reproduced the *ex vivo* phenotypic and functional alterations that were observed in patients with septic shock.

To note, in our model, only full TCR activation through CD3/28 co-activation, and not through CD3 alone, led to phenotypic and functional alterations similar to those observed in patients with septic shock. Previous studies in mice and in humans showed that TCR crosslinking with anti-CD3 Ab in the absence of a co-stimulatory signal induced T-cell anergy [48]. T-cell anergy is a tolerance mechanism in which the lymphocyte is intrinsically functionally inactivated and remains in this hyporeactive state for a long period of time [49]. While we observed that such experimental conditions led to altered T-cell response, in particular in regard to cytokine production capacity, such a model of T-cell anergy did not replicate sepsis-induced T lymphocyte alterations. This suggests that T-cell alterations described in patients with septic shock are not due to an aberrant and incomplete T-cell activation phenomenon but to an overwhelming T-cell activation in a pro-inflammatory context. They are thus probably more related to the phenomenon of exhaustion, as reported in chronic viral infections and cancer. The massive initial T-cell activation in sepsis could have a role in the onset of T-cell exhaustion in a similar but accelerated way. These similarities should be further explored in dedicated experiments (for example, by comparing T-cell transcriptional profiles in these different conditions).

Finally, we showed that IL-7 improved T-cell proliferation in our model, as observed *ex vivo* with T cells from patients with sepsis [10, 15]. This further reinforces the capacity of our model to recapitulate sepsis-induced intrinsic T-cell alterations. To the best of our knowledge, this is the first *ex vivo* model to reproduce intrinsic phenotypic and functional T-cell alterations observed in patients. This model could be used to monitor the efficiency of immuno-adjuvant treatments on T-cell alterations or to investigate pathophysiological mechanisms leading to these alterations in septic shock, such as the role of the altered metabolism that we recently described [15]. Preliminary data are already available in the literature. Patsoukis *et al.* showed that an increased expression of GLUT-1 following TCR activation was inhibited if T cells were pre-activated compared with non-pre-activated T cells [50]. This is very similar to the absence of GLUT-1 induction upon *ex vivo* activation that is observed on T cells from patients with septic shock [15].

The present study has some limitations. In particular, given the small size of this cohort, the differences in percentages of CD127^low^PD-1^high^ population between patients and HVs over time have to be confirmed in a larger number of patients. In addition, further studies should be initiated to better characterize this CD127^low^PD-1^high^ population in patients with septic shock, such as the determination of its transcriptomic profile. It would also be important to study their putative regulatory functions in order to determine whether this subpopulation of CD4^+^ T cells participates in sepsis-induced immune alterations. In addition, several aspects of T-cell dysfunctions remain to be explored in patients with septic shock and in the *ex vivo* model. For example, it would have been interesting to study T-cell phenotype and T helper (Th) polarization in proliferation experiments. Finally, although our results in the *ex vivo* model suggest that TCR activation plays a role in phenotypic and functional alterations observed in septic shock, that does not exclude any other mechanisms that may also contribute to these alterations. For example, the effects induced by other cell types or soluble factors such as cytokines remain to be explored.

## Conclusion

We present here an evaluation of CD127 expression, and more specifically of CD127^low^PD-1^high^ phenotype, in parallel with T-cell activated/exhausted status in patients with septic shock. We also developed an *ex vivo* model of TCR activation-induced T-cell alterations. Our results suggest that T-cell activation may participate in sepsis-induced T lymphocyte exhaustion in patients through the induction of CD127^low^PD-1^high^ T cells. Further comprehension of such activation-induced T-cell exhaustion is now mandatory in order to improve our understanding of the pathophysiology of sepsis-induced T-cell alterations, to develop innovative biomarkers and immuno-adjuvant therapies to restore normal lymphocyte functions in patients. Moreover, the CD127^low^PD-1^high^ T-cell subpopulation should be further investigated given its potential role as a specific marker of T-cell immunosuppression in septic shock.

## Additional files

Additional file 1: **Figure S1.** PD-1 expression in T cells of patients with septic shock in comparison with healthy volunteers (HVs). Cell surface expression of PD-1 was measured on septic shock patients’ CD4^+^ (left panel) and CD8^+^ (right panel) T cells at day 1 (D1, *n* = 15), day 3 (D3, *n* = 21), and day 7 (D7, *n* = 10) after the onset of shock in comparison with HVs (*n* = 20). Results are expressed as median of fluorescence intensity (MFI) on selected T-cell subpopulation. Mann–Whitney tests were used to compare values between patients with septic shock and HVs, *P <0.05. (TIF 79 kb)

Additional file 2: **Figure S2.** Proportions of each node among T cells in patients with septic shock and healthy volunteers (HVs). T-cell phenotype was evaluated by using a SPADE (Spanning-tree Progression Analysis of Density-normalized Events) algorithm based on the expression of different markers measured by flow cytometry on whole blood samples in patients with septic shock at day 3 after the onset of shock (D3, *n* = 17) and in HVs (*n* = 14). Each node represents a cell population with a similar phenotype for the different markers. The proportions of each node are represented among CD4^+^ (left panel) and CD8^+^ (right panel) T cells for patients with septic shock and HVs. Data are presented as Tukey boxplots. Mann–Whitney tests were used to compare values between patients with septic shock and HVs, *P <0.05. (TIF 260 kb)

## Abbreviations

αCD2/3/28: Anti-CD2/CD3/CD28 antibody-coated bead
αCD3: Anti-CD3 antibody-coated bead
αCD3/28: Anti-CD3/CD28 antibody-coated bead
Ab: Antibody
AF: Alexa Fluor
APC: Allophycocyanin
cDNA: Complementary DNA
D: Day
FITC: Fluorescein isothiocyanate
HCV: Hepatitis C virus
HIV: Human immunodeficiency virus
HV: Healthy volunteer
ICU: Intensive care unit
IFN: Interferon
IL-7R: IL-7 receptor
KRO: Krome Orange
MFI: Median of fluorescence intensity
PB: Pacific Blue
PC: Phycoerythrin-cyanin
PCR: Polymerase chain reaction
PE: Phycoerythrin
PMA: Phorbol 12-myristate 13-acetate
sCD127: Soluble CD127
SPADE: Spanning-tree Progression Analysis of Density-normalized Events
TCR: T-cell receptor
TNF: Tumor necrosis factor
Treg: Regulatory T

## References

[1] Singer M, Deutschman CS, Seymour CW, Shankar-Hari M, Annane D, Bauer M (2016). The Third International Consensus Definitions for Sepsis and Septic Shock (Sepsis-3). JAMA.

[2] Angus DC, van der Poll T (2013). Severe Sepsis and Septic Shock. N Engl J Med..

[3] Shankar-Hari M, Phillips GS, Levy ML, Seymour CW, Liu VX, Deutschman CS (2016). Developing a new definition and assessing new clinical criteria for septic shock: For the third international consensus definitions for sepsis and septic shock (sepsis-3). JAMA.

[4] Lagu T, Lindenauer PK, Rothberg MB, Nathanson BH, Pekow PS, Steingrub JS (2011). Development and validation of a model that uses enhanced administrative data to predict mortality in patients with sepsis. Crit Care Med..

[5] Hotchkiss RS, Coopersmith CM, McDunn JE, Ferguson TA (2009). Tilting toward immunosuppression. Nat Med..

[6] Hotchkiss RS, Monneret G, Payen D (2013). Sepsis-induced immunosuppression: from cellular dysfunctions to immunotherapy. Nat Rev Immunol..

[7] Wherry EJ (2011). T cell exhaustion. Nat Immunol..

[8] Boomer JS, To K, Chang KC, Takasu O, Osborne DF, Walton AH (2011). Immunosuppression in patients who die of sepsis and multiple organ failure. JAMA.

[9] Drewry AM, Samra N, Skrupky LP, Fuller BM, Compton SM, Hotchkiss RS (2014). Persistent Lymphopenia after Diagnosis of Sepsis Predicts Mortality. Shock.

[10] Venet F, Foray A-P, Villars-Méchin A, Malcus C, Poitevin-Later F, Lepape A (2012). IL-7 Restores Lymphocyte Functions in Septic Patients. J Immunol..

[11] Chang K, Svabek C, Vazquez-Guillamet C, Sato B, Rasche D, Wilson S (2014). Targeting the programmed cell death 1: programmed cell death ligand 1 pathway reverses T cell exhaustion in patients with sepsis. Crit Care.

[12] Boomer JS, Shuherk-Shaffer J, Hotchkiss RS, Green JM (2012). A prospective analysis of lymphocyte phenotype and function over the course of acute sepsis. Crit Care.

[13] Tomino A, Tsuda M, Aoki R, Kajita Y, Hashiba M, Terajima T (2017). Increased PD-1 Expression and Altered T Cell Repertoire Diversity Predict Mortality in Patients with Septic Shock: A Preliminary Study. PLoS One.

[14] Unsinger J, McGlynn M, Kasten KR, Hoekzema AS, Watanabe E, Muenzer JT (2010). IL-7 promotes T cell viability, trafficking, and functionality and improves survival in sepsis. J Immunol.

[15] Venet F, Demaret J, Blaise BJ, Rouget C, Girardot T, Idealisoa E (2017). IL-7 Restores T Lymphocyte Immunometabolic Failure in Septic Shock Patients through mTOR Activation. J Immunol..

[16] Francois B, Jeannet R, Daix T, Walton AH, Shotwell MS, Unsinger J, et al. Interleukin-7 restores lymphocytes in septic shock: the IRIS-7 randomized clinical trial. JCI Insight. 2018;3. 10.1172/jci.insight.98960 PMC5922293 29515037

[17] Minton K (2002). IL-7 fine-tunes T-cell homeostasis. Nat Rev Immunol..

[18] Lundström W, Fewkes NM, Mackall CL (2012). IL-7 in human health and disease. Semin Immunol..

[19] Paiardini M, Cervasi B, Albrecht H, Muthukumar A, Dunham R, Gordon S (2005). Loss of CD127 Expression Defines an Expansion of Effector CD8+ T Cells in HIV-Infected Individuals. J Immunol..

[20] Golden-Mason L, Burton JR, Castelblanco N, Klarquist J, Benlloch S, Wang C (2006). Loss of IL-7 receptor alpha-chain (CD127) expression in acute HCV infection associated with viral persistence. Hepatol Baltim Md..

[21] Vudattu NK, Magalhaes I, Schmidt M, Seyfert-Margolis V, Maeurer MJ (2007). Reduced numbers of IL-7 receptor (CD127) expressing immune cells and IL-7-signaling defects in peripheral blood from patients with breast cancer. Int J Cancer.

[22] McKinney EF, Lee JC, Jayne DR, Lyons PA, Smith KG (2015). T-cell exhaustion, co-stimulation and clinical outcome in autoimmunity and infection. Nature.

[23] Radziewicz H, Ibegbu CC, Fernandez ML, Workowski KA, Obideen K, Wehbi M (2007). Liver-Infiltrating Lymphocytes in Chronic Human Hepatitis C Virus Infection Display an Exhausted Phenotype with High Levels of PD-1 and Low Levels of CD127 Expression. J Virol..

[24] Matsuzaki J, Gnjatic S, Mhawech-Fauceglia P, Beck A, Miller A, Tsuji T (2010). Tumor-infiltrating NY-ESO-1-specific CD8+ T cells are negatively regulated by LAG-3 and PD-1 in human ovarian cancer. Proc Natl Acad Sci U S A.

[25] Bengsch B, Seigel B, Ruhl M, Timm J, Kuntz M, Blum HE (2010). Coexpression of PD-1, 2B4, CD160 and KLRG1 on exhausted HCV-specific CD8+ T cells is linked to antigen recognition and T cell differentiation. PLoS Pathog..

[26] Trautmann L, Janbazian L, Chomont N, Said EA, Gimmig S, Bessette B (2006). Upregulation of PD-1 expression on HIV-specific CD8+ T cells leads to reversible immune dysfunction. Nat Med..

[27] Levy MM, Fink MP, Marshall JC, Abraham E, Angus D, Cook D (2003). 2001 SCCM/ESICM/ACCP/ATS/SIS International Sepsis Definitions Conference. Crit Care Med.

[28] Peronnet E, Venet F, Maucort-Boulch D, Friggeri A, Cour M, Argaud L (2017). Association between mRNA expression of CD74 and IL10 and risk of ICU-acquired infections: a multicenter cohort study. Intensive Care Med..

[29] Suetens C, Morales I, Savey A, Palomar M, Hiesmayr M, Lepape A (2007). European surveillance of ICU-acquired infections (HELICS-ICU): methods and main results. J Hosp Infect..

[30] Demaret J, Walencik A, Jacob M-C, Timsit J-F, Venet F, Lepape A (2013). Inter-laboratory assessment of flow cytometric monocyte HLA-DR expression in clinical samples. Cytometry B Clin Cytom..

[31] Venet F, Chung C-S, Kherouf H, Geeraert A, Malcus C, Poitevin F (2009). Increased circulating regulatory T cells (CD4+CD25+CD127−) contribute to lymphocyte anergy in septic shock patients. Intensive Care Med..

[32] Schwartz RH (1990). A cell culture model for T lymphocyte clonal anergy. Science.

[33] Qiu P, Simonds EF, Bendall SC, Gibbs KD, Bruggner RV, Linderman MD (2011). Extracting a cellular hierarchy from high-dimensional cytometry data with SPADE. Nat Biotechnol..

[34] Yin Y, Mitson-Salazar A, Prussin C (2015). Detection of Intracellular Cytokines by Flow Cytometry. Curr Protoc Immunol..

[35] Letessier W, Demaret J, Gossez M, Allam C, Venet F, Rimmelé T (2018). Decreased intra-lymphocyte cytokines measurement in septic shock patients: A proof of concept study in whole blood. Cytokine.

[36] Poujol F, Monneret G, Friggeri A, Rimmelé T, Malcus C, Poitevin-Later F (2014). Flow cytometric evaluation of lymphocyte transformation test based on 5-ethynyl-2’deoxyuridine incorporation as a clinical alternative to tritiated thymidine uptake measurement. J Immunol Methods.

[37] Bisset LR, Lung TL, Kaelin M, Ludwig E, Dubs RW (2004). Reference values for peripheral blood lymphocyte phenotypes applicable to the healthy adult population in Switzerland. Eur J Haematol..

[38] Monneret G, Venet F (2016). Sepsis-induced immune alterations monitoring by flow cytometry as a promising tool for individualized therapy. Cytometry B Clin Cytom..

[39] Legat A, Speiser DE, Pircher H, Zehn D, Fuertes Marraco SA (2013). Inhibitory Receptor Expression Depends More Dominantly on Differentiation and Activation than “Exhaustion” of Human CD8 T Cells. Front Immunol..

[40] Duraiswamy J, Ibegbu CC, Masopust D, Miller JD, Araki K, Doho GH (2011). Phenotype, function, and gene expression profiles of programmed death-1(hi) CD8 T cells in healthy human adults. J Immunol.

[41] Motamedi M, Xu L, Elahi S (2016). Correlation of transferrin receptor (CD71) with Ki67 expression on stimulated human and mouse T cells: The kinetics of expression of T cell activation markers. J Immunol Methods.

[42] Chung K-P, Chang H-T, Lo S-C, Chang L-Y, Lin S-Y, Cheng A (2015). Severe lymphopenia is associated with elevated plasma interleukin-15 levels and increased mortality during severe sepsis. Shock.

[43] Hotchkiss RS, Moldawer LL (2014). Parallels between cancer and infectious disease. N Engl J Med..

[44] Unsinger J, C-AD B, McDonough J, Morre M, Prakash PS, Caldwell CC (2012). Interleukin-7 ameliorates immune dysfunction and improves survival in a 2-hit model of fungal sepsis. J Infect Dis..

[45] Ahmadzadeh M, Johnson LA, Heemskerk B, Wunderlich JR, Dudley ME, White DE (2009). Tumor antigen-specific CD8 T cells infiltrating the tumor express high levels of PD-1 and are functionally impaired. Blood.

[46] Borken F, Markwart R, Requardt RP, Schubert K, Spacek M, Verner M (2017). Chronic Critical Illness from Sepsis Is Associated with an Enhanced TCR Response. J Immunol..

[47] Schwulst SJ, Muenzer JT, Chang KC, Brahmbhatt TS, Coopersmith CM, Hotchkiss RS (2008). Lymphocyte phenotyping to distinguish septic from nonseptic critical illness. J Am Coll Surg..

[48] Nguyen DD, Beck L, Spiegelberg HL (1995). Anti-CD3-Induced Anergy in Cloned Human Th0, Th1, and Th2 Cells. Cell Immunol..

[49] Schwartz RH (2003). T cell anergy. Annu Rev Immunol..

[50] Patsoukis N, Bardhan K, Chatterjee P, Sari D, Liu B, Bell LN (2015). PD-1 alters T-cell metabolic reprogramming by inhibiting glycolysis and promoting lipolysis and fatty acid oxidation. Nat Commun..

