# Supplementary figures and images for: TCR activation mimics CD127lowPD-1high phenotype and functional alterations of T lymphocytes from septic shock patients
Source: Crit Care. 2019 Apr 17;23:131. doi: 10.1186/s13054-018-2305-5 (PMC6472012; doi:10.1186/s13054-018-2305-5)

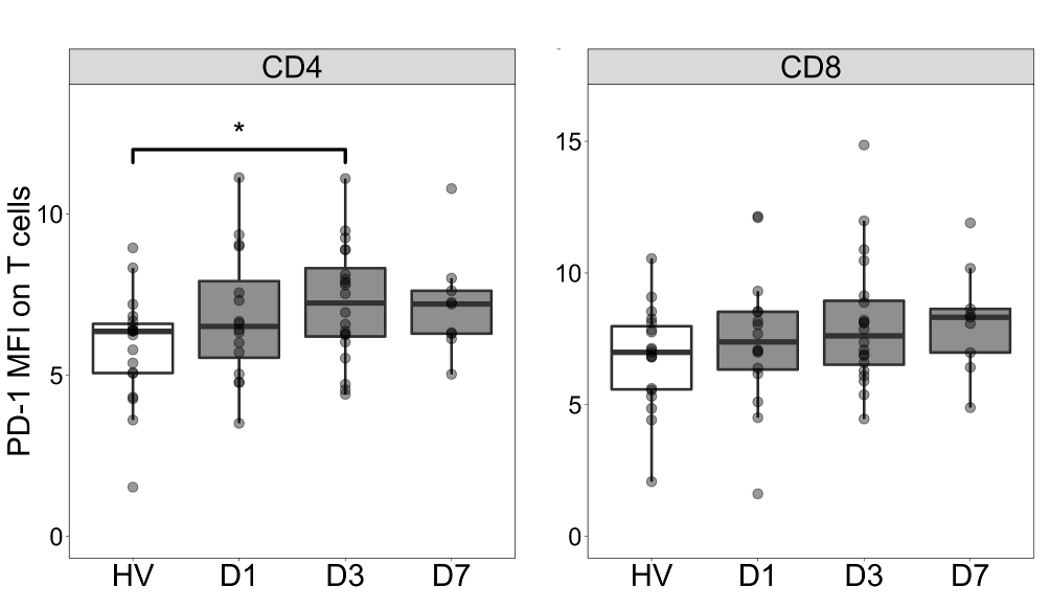

Supplement: Supplementary file 1 — Figure S1. PD-1 expression in T cells of patients with septic shock in comparison with healthy volunteers (HVs). Cell surface expression of PD-1 was measured on septic shock patients’ CD4+ (left panel) and CD8+ (right panel) T cells at day 1 (D1, n = 15), day 3 (D3, n = 21), and day 7 (D7, n = 10) after the onset of shock in comparison with HVs (n = 20). Results are expressed as median of fluorescence intensity (MFI) on selected T-cell subpopulation. Mann–Whitney tests were used to compare values between patients with septic shock and HVs, *P <0.05. (TIF 79 kb) [file 13054_2018_2305_MOESM1_ESM.tif]

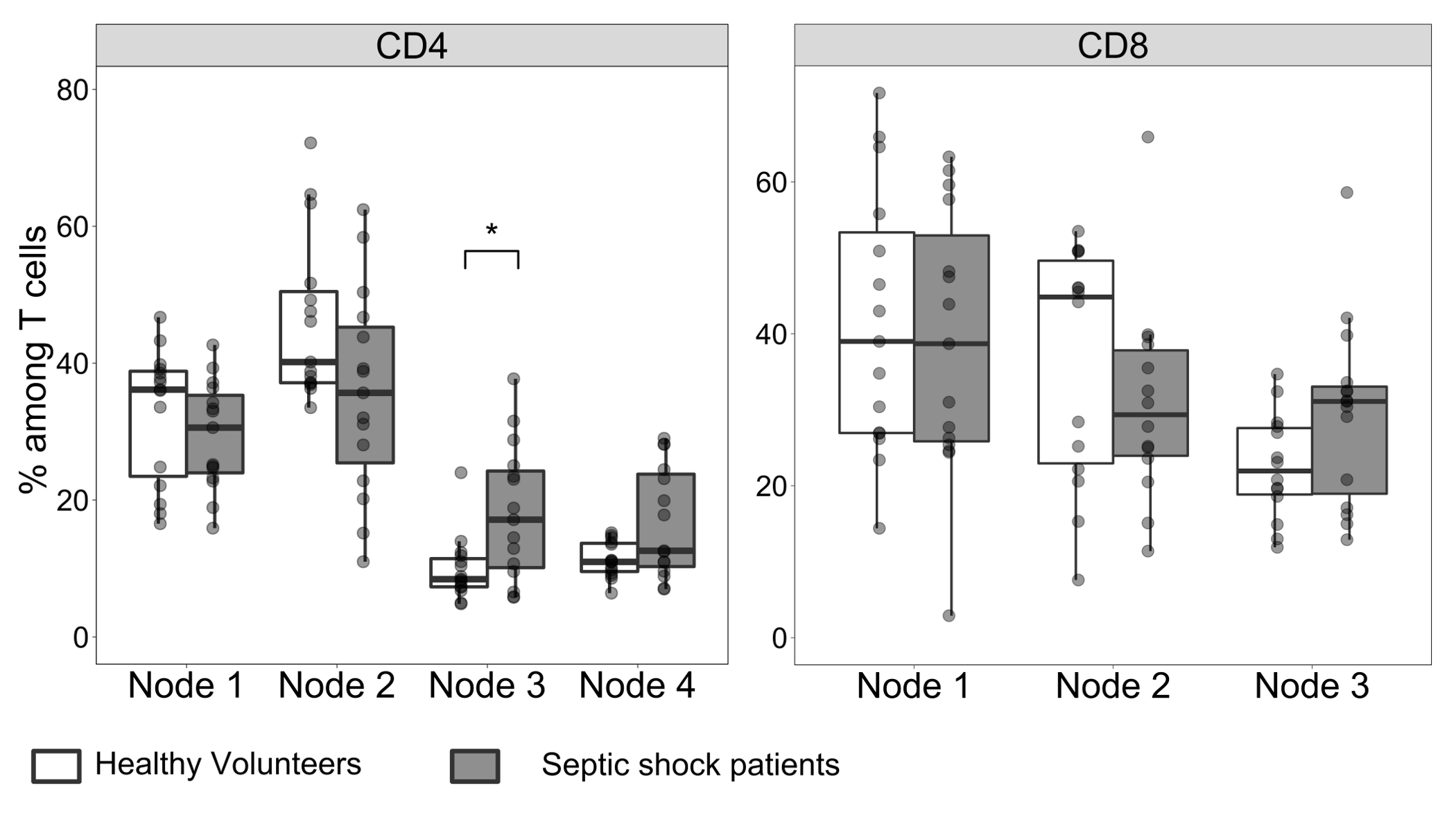

Supplement: Supplementary file 2 — Figure S2. Proportions of each node among T cells in patients with septic shock and healthy volunteers (HVs). T-cell phenotype was evaluated by using a SPADE (Spanning-tree Progression Analysis of Density-normalized Events) algorithm based on the expression of different markers measured by flow cytometry on whole blood samples in patients with septic shock at day 3 after the onset of shock (D3, n = 17) and in HVs (n = 14). Each node represents a cell population with a similar phenotype for the different markers. The proportions of each node are represented among CD4+ (left panel) and CD8+ (right panel) T cells for patients with septic shock and HVs. Data are presented as Tukey boxplots. Mann–Whitney tests were used to compare values between patients with septic shock and HVs, *P <0.05. (TIF 260 kb) [file 13054_2018_2305_MOESM2_ESM.tif]
